# Structures of L-BC virus and its open particle provide insight into *Totivirus* capsid assembly

**DOI:** 10.1038/s42003-022-03793-z

**Published:** 2022-08-20

**Authors:** Danyil Grybchuk, Michaela Procházková, Tibor Füzik, Aleksandras Konovalovas, Saulius Serva, Vyacheslav Yurchenko, Pavel Plevka

**Affiliations:** 1 Central European Institute of Technology, Masaryk University, 62500 Brno, Czech Republic; 2 Department of Biochemistry and Molecular Biology, Vilnius University, 10257 Vilnius, Lithuania; 3 Life Science Research Centre, Faculty of Science, University of Ostrava, 71000 Ostrava, Czech Republic

**Keywords:** Cryoelectron microscopy, Virology

## Abstract

L-BC virus persists in the budding yeast *Saccharomyces cerevisiae*, whereas other viruses from the family *Totiviridae* infect a diverse group of organisms including protists, fungi, arthropods, and vertebrates. The presence of totiviruses alters the fitness of the host organisms, for example, by maintaining the killer system in yeast or increasing the virulence of *Leishmania guyanensis*. Despite the importance of totiviruses for their host survival, there is limited information about *Totivirus* structure and assembly. Here we used cryo-electron microscopy to determine the structure of L-BC virus to a resolution of 2.9 Å. The L-BC capsid is organized with icosahedral symmetry, with each asymmetric unit composed of two copies of the capsid protein. Decamers of capsid proteins are stabilized by domain swapping of the C-termini of subunits located around icosahedral fivefold axes. We show that capsids of 9% of particles in a purified L-BC sample were open and lacked one decamer of capsid proteins. The existence of the open particles together with domain swapping within a decamer provides evidence that *Totiviridae* capsids assemble from the decamers of capsid proteins. Furthermore, the open particles may be assembly intermediates that are prepared for the incorporation of the virus (+) strand RNA.

## Abstract (web summary)

A 2.9 Å resolution structure of the L-BC virus provides insight into the contacts between capsid proteins and the mechanism of capsid assembly.

## Introduction

*Totiviridae* is a family of viruses with monopartite double-stranded RNA (dsRNA) genomes, which contain two open reading frames encoding the capsid protein and the RNA-dependent RNA-polymerase (RdRp). Members of this family infect fungi (genera *Totivirus* and *Victorivirus*) and unicellular protists (genera *Trichomonasvirus*, *Giardiavirus*, and *Leishmaniavirus*). In addition, several as-yet unclassified totiviruses were identified in arthropods^1,2^ and vertebrates^3,4^. Most totiviruses belong to the ecological group of mycoviruses that persist as complete virions in the cytoplasm of infected cells and spread through cell division or contact with the cytoplasm^5^. In contrast, the arthropod totiviruses and *Giardia lamblia virus* were shown to be capable of extracellular transmission^6–8^.

Two totiviruses are known to infect yeasts: *Saccharomyces cerevisiae virus L-A* (L-A) and *Saccharomyces cerevisiae virus L-BCLa* (L-BC)^9^. Wild strains of yeasts occasionally also contain M particles, which carry the sole gene for a toxin-antitoxin system and ensure retention of the virus similarly to bacterial plasmid addiction systems^10,11^. The M-viruses do not encode capsid proteins and RdRp, but are maintained by the L-A helper virus. There are at least four types of M-viruses (K1, K2, Klus, and K28 killer phenotypes), each maintained by a specific L-A virus that competes with the others in a given yeast population^12^. This proverbial tug of war results in a co-evolution of L-A and M viruses^13^. The L-BC virus accompanies L-A in at least 50% of natural yeast populations and co-evolves with it^13,14^. However, it is possible to generate a L-BC-positive/L-A-negative yeast strain. In such a strain, the L-A-associated killer phenotype is lost, which indicates that L-BC cannot serve as a helper virus for the toxin-expressing M-virus^15^. Various stable L-A/L-BC combinations can be obtained by the cytoplasmic mixing of laboratory yeast strains, suggesting that there is no interdependency between naturally co-occurring L-A and L-BC viruses^14^.

Capsid structures of six members of the family *Totiviridae* and two arthropod totiviruses were determined^7,16–23^. The capsids of totiviruses are organized with icosahedral symmetry and have a diameter of 40 nm. The icosahedral asymmetric unit is formed by two copies of a capsid protein. The arrangement of a capsid with two subunits in an icosahedral asymmetric unit is a feature shared by many viruses with dsRNA genomes that infect bacteria and eukaryotes. Capsids of dsRNA viruses either consist of a single protein layer (toti-, partiti-, chryso-, megabirna- and quadri- and picobirnaviruses) or possess one or two additional layers with more complex structure (cystoviruses and reoviruses with the exception of a single-shelled *Cypovirus*)^24,25^. Structural studies revealed polymerase in the virions of several members of the families *Reoviridae*^26–31^ and *Cystoviridae*^32,33^. The incorporation of RdRp into a capsid is a crucial step in dsRNA virus assembly as it enables the replication and transcription of the viral genome inside the virion, avoiding the host antiviral response^34,35^. Open reading frames of capsids and RdRps of most totiviruses that infect protists and fungi overlap. The RdRp is expressed as a C-terminal extension of the capsid protein thanks to a ribosomal frameshifting sequence between the two genes^36–39^. In L-A virus, the efficiency of the ribosomal frameshifting is 1.9%, which results in the incorporation of two RdRps per virion on average^40^. A secondary structure at the 3′-end of the (+)RNA strand of the L-A virus genome is recognized by the RNA-binding domain of the RdRp^41–43^ and ensures genome encapsidation^44^.

The RdRps of totiviruses produce RNA molecules that are not capped at the 5′-ends^45^. Therefore, the virus RNA molecules are not recognized by the host translation factors and may be targeted for degradation by host cell RNA quality control systems^46^. Particles of L-A and L-BC were reported to de-cap cellular mRNAs, which may serve as decoys to overload cellular RNA-degradation machinery^47,48^. Furthermore, there is evidence that both L-A and L-BC have a unique cap-snatching mechanism that transfers eukaryotic cap 0 (m7G) to viral transcripts. First, m7G from a host mRNA is covalently bound to a histidine from a capsid protein and then transferred to a viral mRNA^49,50^.

In this study we describe the structure of the L-BC capsid, determined to a resolution of 2.9 Å. The putative mRNA decapping site in the capsid protein of L-BC is homologous to that of L-A virus. We describe domain swap stabilizing interactions between capsid proteins of L-BC that form a decamer. Furthermore, we identified an open particle of L-BC that lacks one decamer of capsid proteins. These observations indicate that the decamers of capsid proteins are the building blocks for the L-BC capsid. We discuss the stochastic incorporation of capsid-RdRp fusion protein into the L-BC capsid, and pinpoint the role of the −1 ribosome frameshifting site in successful virion assembly.

## Results and discussion

### Structure of L-BC virus

**Fig. 1:**
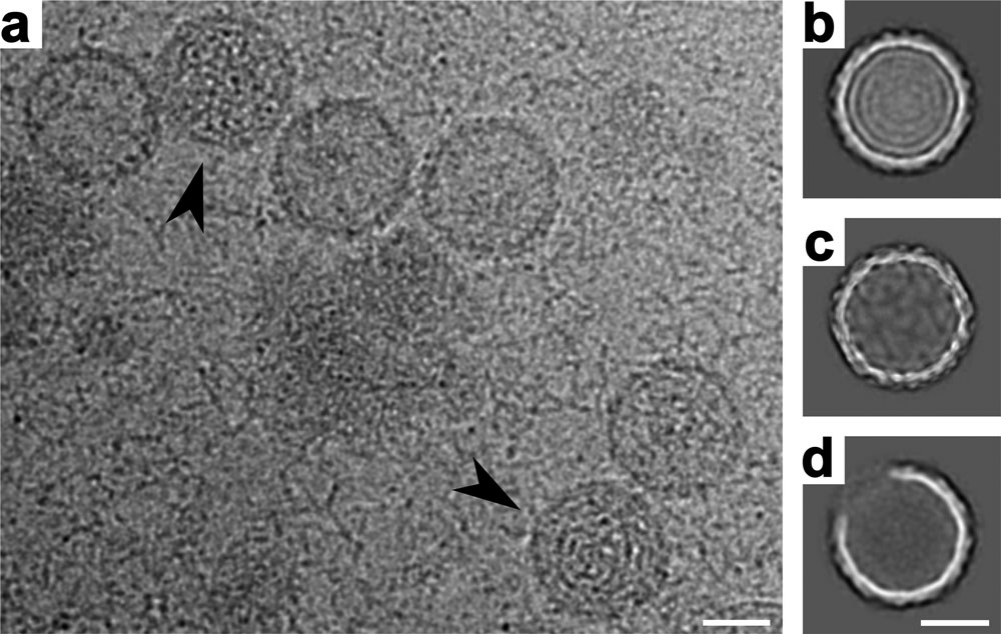
Preparation of L-BC virus isolated from Saccharomyces cerevisiae contained full, empty, and open particles. **a** A selected cryo-electron micrograph showing full (arrowheads) and empty virus particles. The hair-like features in the background are probably formed by virus RNA released as a result of particle damage, and the gray blobs might be cell debris. Two-dimensional class averages of L-BC virion (**b**), empty (**c**), and open (**d**) particles. Scale bar 200 Å.

**Fig. 2:**
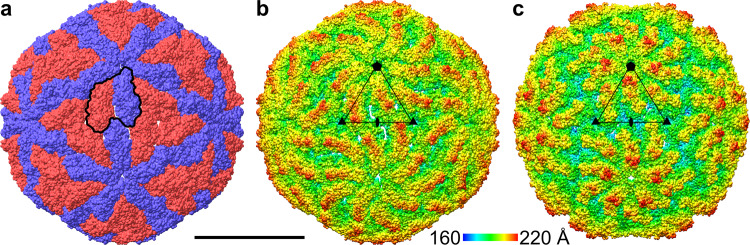
Comparison of virion structures of L-BC and L-A viruses. **a**, **b** Molecular surface representations of L-BC virus. Scale bar 200 Å. **a** Particle surface is colored according to type of subunit within icosahedral asymmetric unit: A in blue and B in red. The borders of a selected icosahedral asymmetric unit are highlighted with a black outline. **b** Particle surface is rainbow-colored based on distance from particle center. Positions of selected fivefold, threefold, and twofold symmetry axes are indicated with pentagon, triangles, and oval, respectively. **c** Rainbow-colored molecular surface representation of L-A virus (PDB 1M1C). The rainbow scale shows the distance of the molecular surface from the particle center in Ångstroms.

**Fig. 3:**
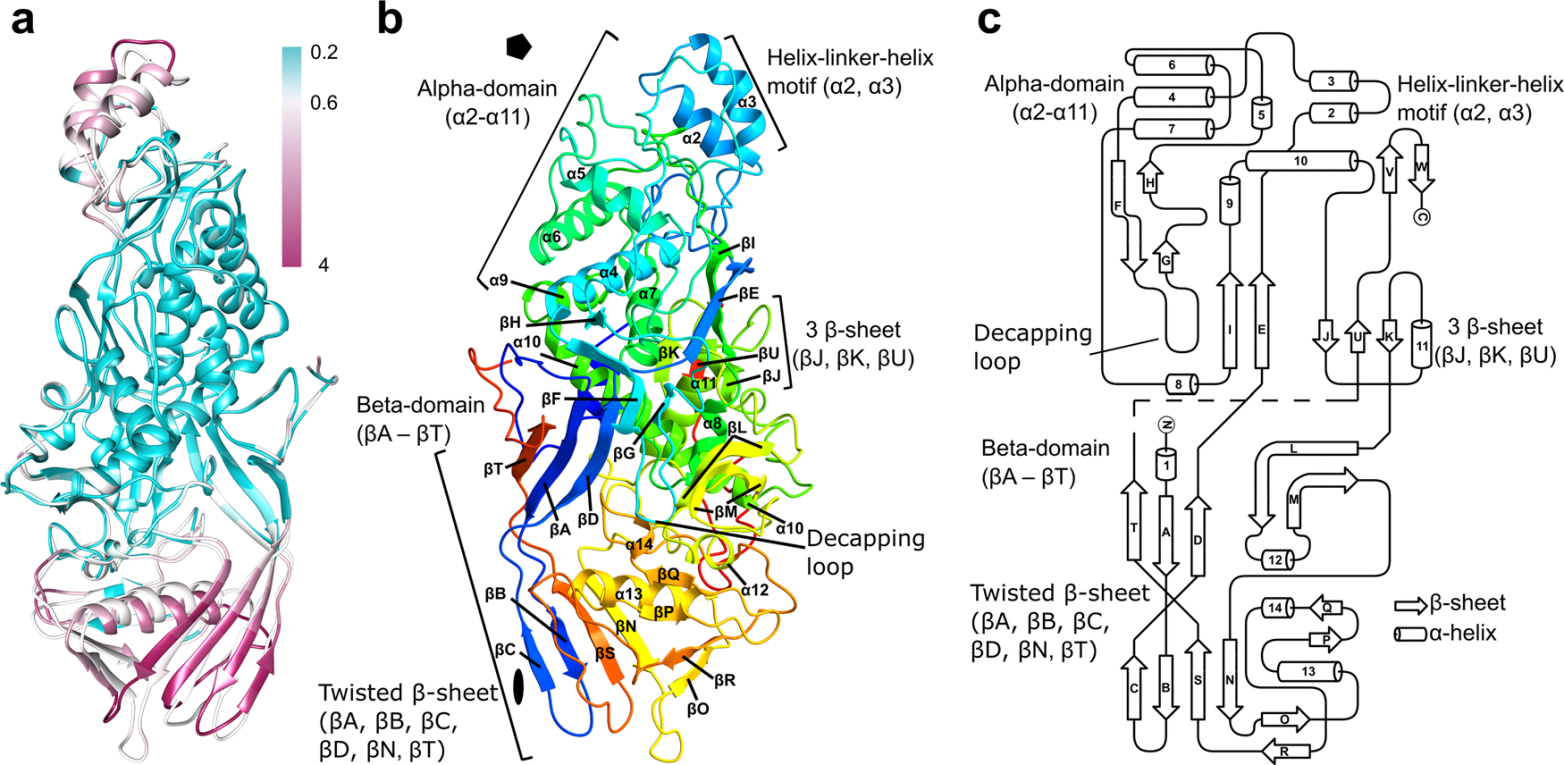
Structure of L-BC capsid protein. **a** Comparison of structures of L-BC A and B subunits. The B subunit is colored based on the RMSD difference in the positions of atoms forming the corresponding residues of A and B subunits. The A subunit is colored the same as B, except for residues with RMSD larger than 0.6 Å, which are white. **b** Cartoon representation of A subunit rainbow-colored from N-terminus in blue to C-terminus in red. The secondary structure elements are labeled. The positions of twofold and fivefold icosahedral symmetry axes are indicated with an oval and pentagon, respectively. **c** Two-dimensional topology representation of secondary structure elements of A subunit.

**Fig. 4:**
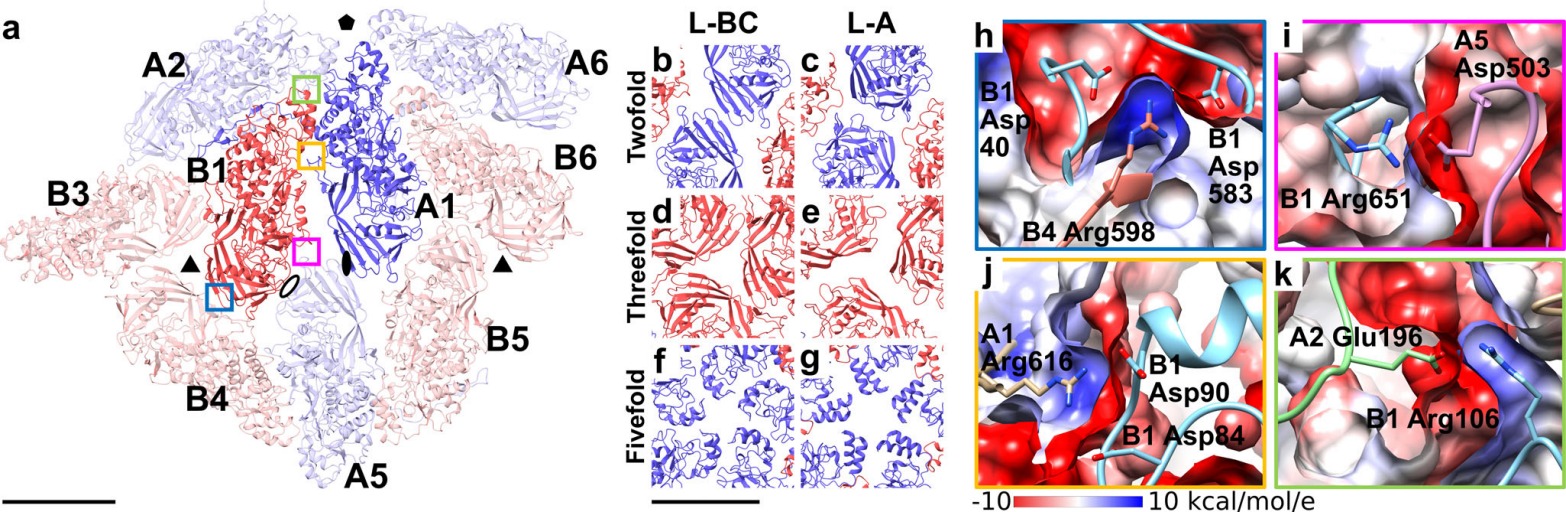
Inter-subunit interactions in L-BC capsid. **a** Cartoon representation of L-BC capsid proteins forming an icosahedral asymmetric unit (bright colors) with all their interaction partners within the capsid (pale colors). View from outside the capsid. Scale bar 50 Å. A subunits are shown in blue and B subunits are shown in red. Subunits belonging to different icosahedral units are indicated with numbers. Positions of selected icosahedral symmetry axes are indicated: twofold - oval, threefold - triangle, fivefold - pentagon, and quasi-twofold - empty oval. **b**–**g** Comparisons of intersubunit contacts around icosahedral symmetry axes of L-BC and L-A viruses. The coloring scheme is the same as in **a**. Scale bar 50 Å. **h**–**k** Details of interactions between capsid proteins of L-BC. The color of the frame of each panel corresponds to that of boxed areas in **a**. The capsid proteins are shown in cartoon representation with side chains of selected residues depicted in stick-representation. The side chains are colored according to the atom type: oxygen in red and nitrogen in blue. Molecular surfaces colored according to electrostatic potential are shown to demonstrate the compatibility of interacting interfaces.

Two-dimensional classification of particle images from a purified L-BC virus sample identified 87% empty particles, 9% open particles, and 4% genome-containing virions (Fig. 1). The structure of the empty particle was determined to a resolution of 2.9 Å, whereas those of open particles and virions to 10 and 3.7 Å, respectively (Table 1 and Supplementary Fig. 1). The capsid of L-BC is organized with icosahedral symmetry with two subunits of capsid protein, named A and B, forming the icosahedral asymmetric unit (Figs. 2, 3). The two subunits in the icosahedral asymmetric unit have non-equivalent binding environments. The A subunit connects twofold and fivefold axes of icosahedral symmetry of the capsid, whereas B subunits interact around the icosahedral threefold axes (Figs. 2, 4a). Overall, the capsid can be imagined as built from twelve decamers, each composed of five dimers of the capsid protein (Fig. 2a). This arrangement of subunits, without quasi-equivalence of capsid protein interactions, is exceptional among icosahedral viruses, however, it is a common feature of viruses that replicate their dsRNA genomes inside capsids^24,51^.

**Table 1:** Cryo-EM structure quality indicators.

Data collection and processing	Empty particle	Full virion	Open particle C5	Open particle C1
Magnification	130,000	130,000	130,000	130,000
Voltage (kV)	300	300	300	300
Exposure (e^–^.Å^–2^)	36	36	36	36
Pixel size (Å)	1.079	1.079	1.079	1.079
Symmetry	I4	I4	C5	C1
Init. No of particles	72705	72705	72705	72705
Fin. No of particles	17702	1748	1120	1120
Map resolution (Å)	2.9	3.7	10	12
FSC threshold	0.143	0.143	0.143	0.143
Refinement				
Software	RELION 3.1	RELION 3.1	RELION 3.1	RELION 3.1
Initial model	*de novo*	Empty particle	Empty particle	Empty particle
Model composition and quality				
Atoms (except hydrogens)	10414^a^	10414^a^	112191^b^	112191^b^
Residues	1316^a^	1316^a^	14181^b^	14181^b^
B-factors				
Protein (asymmetric unit)	30.01	65.16	N/A^c^	N/A^c^
R.M.S. deviations				
Bond length (Å)	0.0022	0.0022^d^	0.0022^d^	0.0022^d^
Bond angles (°)	1.2455	1.2455^d^	1.2455^d^	1.2455^d^
Validation				
MolProbity score (percentile)	1.08 (^100th^)	1.08 (^100th^)^d^	1.08 (^100th^)^d^	1.08 (^100th^)^d^
ClashScore (percentile)	0.34 (^100th^)	0.34 (^100th^)^d^	0.34 (^100th^)^d^	0.34 (^100th^)^d^
Poor Rotamers (%)	0.79	0.79^d^	0.79^d^	0.79^d^
Ramachandran plot				
Outliers (%)	0	0^d^	0^d^	0^d^
Favored (%)	92.68	92.68^d^	92.68^d^	92.68^d^
Accession codes				
PDB	7QWX	7QWZ	7ZUF	7ZTS
EMDB	EMD-14194	EMD-14195	EMD-14975	EMD-14963

^a^Number of atoms or residues in icosahedral asymmetric unit.

^b^Number of atoms or residues in eleven icosahedral asymmetric units forming one-fifth of the particle. The icosahedral asymmetric unit forming the border of the opening contains fewer resolved residues than those from the remainder of the capsid.

^c^B-factors were not refined because of the limited resolution of the cryo-EM reconstruction.

^d^Protein geometry was refined against the reconstruction of the empty particle. The structures of capsid proteins from the empty particle were fitted into the other reconstructions.

The capsid of L-BC is similar to those of L-A virus and other totiviruses of protists and fungi (Figs. 2, 4a)^16,18–21^. In L-A virus, the largest interface between A and B subunits has a buried surface area of 1500 Å^2^, whereas the second largest interface has an area of 1400 Å^2^^21^^,^. In contrast, in L-BC both of the corresponding interfaces have buried surface areas of 1350 Å^2^ (Supplementary Table 1). The arrangement of A and B subunits in the icosahedral asymmetric unit of L-BC was selected to reflect the largest interface in L-A virus (Fig. 4a). Virions of L-BC and L-A have diameters of 38.5 and 37.0 nm, respectively (Fig. 2b, c). The L-A virus has higher protrusions around fivefold symmetry axes and broader depressions around twofold symmetry axes (Fig. 2b, c). Overall, the L-BC virus has a smoother capsid surface than L-A. The capsid proteins of L-BC form elevated ridges around threefold axes, whereas those of L-A protrude around fivefold symmetry axes (Fig. 2b, c). The thickness of both L-BC and L-A capsids is 6 nm. The volume of capsid cavity is 18,000 nm^3^ for L-BC and 17,000 nm^3^ for L-A.

The capsid of the L-BC virion contains pores positioned at five- and threefold symmetry axes with diameters of 12 and 9 Å, respectively (Fig. 4b–g). Furthermore, there are 4- to 7-nm-wide crevices in clefts between capsid proteins. Based on homology with reoviruses, the pores at fivefold symmetry axes were speculated to serve as channels for the release of transcripts from *Totivirus* capsids^16,31^. All the pores in the L-BC capsid can serve as entrances for ribonucleotides, which are required for genome replication. The inner surfaces of capsids and fivefold pores of fungal dsRNA viruses are predominantly negatively charged, probably to facilitate the movements of RNA required for its transcription and release from the capsid^24,52^. The inner capsid surfaces of L-BC and L-A are also negatively charged, however, the fivefold pore of L-BC includes the residues Lys100, Arg105, and Arg106, and is positively charged, whereas that of L-A virus is neutral (Supplementary Fig. 2). The positive charge inside the fivefold pore of L-BC capsid may direct the nascent mRNA transcript for release.

The capsid cavity of the L-BC virion contains a spherical shell and a sphere of density corresponding to the icosahedrally averaged dsRNA genome (Supplementary Fig. 3). Similar to *Trichomonas vaginalis virus 2*^22^, the outermost shell of the genome density is separated from the inner capsid surface by 2 nm. The separation is probably caused by the repulsion between the genome and predominantly negatively charged inner capsid surface (Supplementary Fig. 2). The electrostatic repulsion between the capsid and genome probably facilitates the movements of the RNA during its transcription inside the virion.

### Structure and interactions of L-BC capsid proteins

The capsid proteins of totiviruses can be divided into alpha- and beta-domains (Fig. 3). The alpha-domain of L-BC capsid protein is formed by twelve alpha-helices (ɑ2-13). Alpha-helices ɑ2 and ɑ3 form a helix-turn-helix motif at the surface of the capsid protein (Fig. 3b, c). The beta domain is composed of a twisted beta-sheet of four N-terminal strands (βA-D) and three C-terminal strands (βN, βS, βT) (Fig. 3b, c). The alpha-helical cores of L-BC A and B subunits have nearly identical structures, which can be superimposed with RMSD of 0.6 Å (Fig. 3a). Most of the conformational differences between the A and B subunits are due to their accommodation to non-quasi-equivalent environments in the capsid. The tips of beta-domains of A subunits interact with each other across twofold symmetry axes (Fig. 4b, d). In contrast, the tips of beta domains of B subunits bind each other around the threefold symmetry axis (Fig. 4b, d). The helix-turn-helix motifs of A subunits are positioned adjacent to the fivefold symmetry axis, whereas those of B subunits are inserted between alpha-domains of two neighboring A subunits within a decamer (Fig. 4a).

The interfaces among L-BC capsid proteins are stabilized by electrostatic interactions and topological surface complementarity (Fig. 4h–k). Within the asymmetric unit the electrostatic interactions are mediated by Lys16, Arg616, and Lys262 of the A subunit and Asp70, Asp71, Asp84, Asp90 Glu117, and Glu635 of the B subunit. Arg616 of A1 forms salt bridges with Asp90 and Asp84 of B1 (Fig. 4j) and Lys262 of A forms a salt bridge with Glu117 of B. The electrostatic interactions between the asymmetric units are formed by Lys100, Arg106, Gln273, and Lys337 of B1 subunit and Asp88, Glu196, and Glu384 of A2 subunit and also include salt bridges between Lys100 of B1 and Asp88 of A2, as well as that between Arg106 of B1 and Glu196 of A2 (Fig. 4k).

The interactions between decamers of capsid proteins include the stacking of beta-sheets βB, βC, βN, βS of A subunits across a twofold symmetry axis of the capsid. The interface includes intermolecular H-bonds between antiparallel βC strands (residues Asp41-Lys46) of A subunits. As a result, an eight-stranded beta-sheet is formed (Fig. 4b). The contacts around the threefold symmetry axes include the interactions of Arg598 with Asp40 (salt bridge) and Asp583 (hydrogen bond) from a neighboring B subunit (Fig. 4h). Most of the interaction interface between A and B subunits related by a quasi-twofold symmetry axis is mediated by hydrophobic residues Val508, Leu534, Val535, Ala563, and Leu565, all contributed by both subunits (Supplementary Fig. 4). Furthermore, Asp503 of the A subunit and Arg651 of the B subunit, related by a quasi-twofold axis, form a salt bridge positioned close to the icosahedral twofold axis (Fig. 4i).

### Domain swapping between A subunits within a decamer

**Fig. 5:**
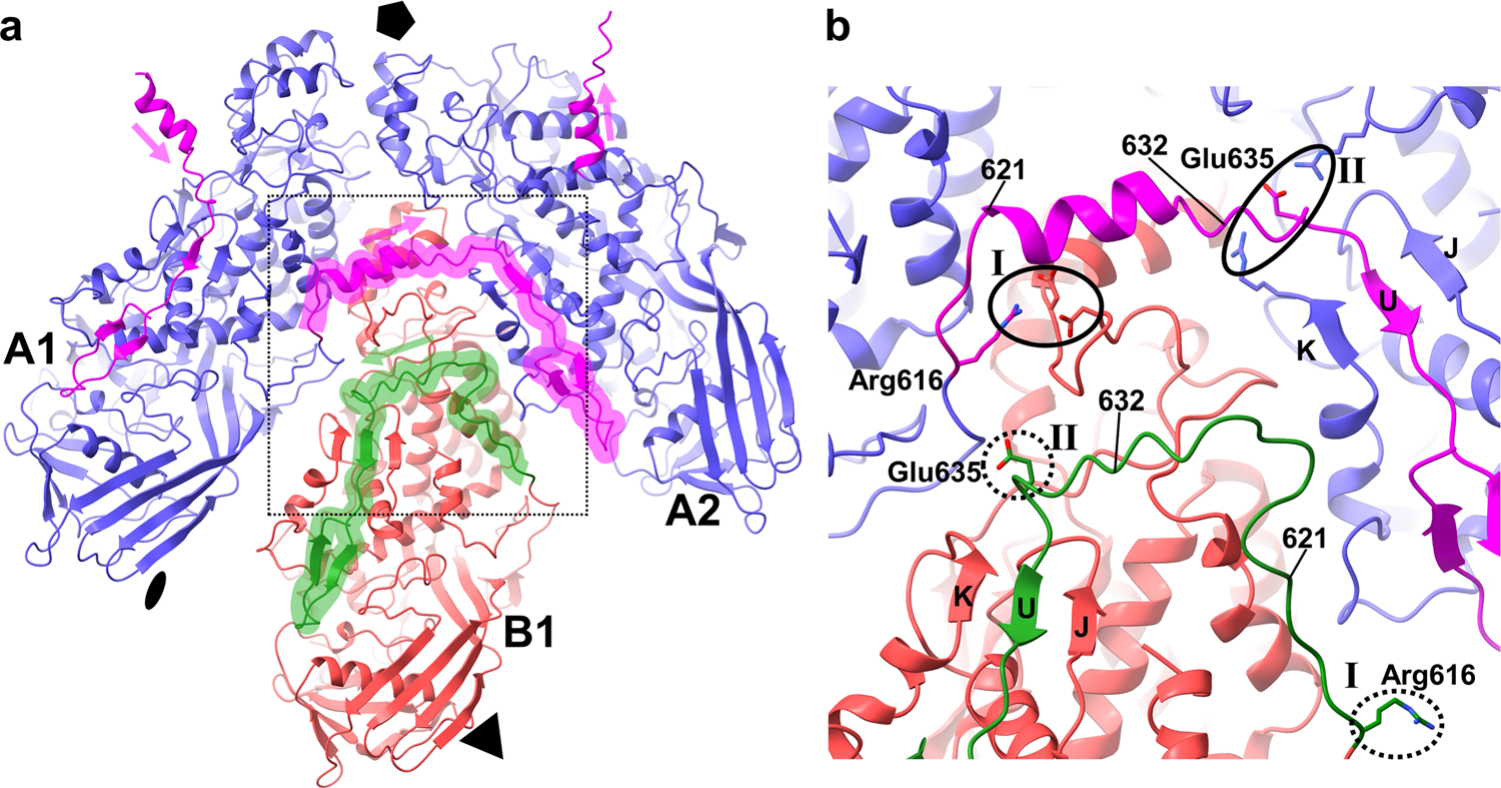
Interactions in L-BC capsid are mediated by domain swapping between A but not B subunits. **a** Cartoon representation of L-BC icosahedral asymmetric unit and A subunit from a neighboring icosahedral asymmetric unit related by fivefold rotation. The subunits are viewed from the inside of the particle. Subunits are distinguished by color: A in blue and B in red. The C-terminus of the A subunit, colored and highlighted in magenta, participates in domain swapping between adjacent A subunits. Domain swaps propagate around the fivefold symmetry axis counterclockwise (magenta arrows). The C-terminus of the B subunit, colored and highlighted in green, remains within the bulk of the protein. **b** Detail of area outlined by dashed square in **a**. The domain swap between A subunits is stabilized by salt bridges at two positions denoted I and II and indicated with black ovals. Side chains of residues forming the salt bridges are shown in stick representation. The corresponding residues from the C-terminus of the B subunit are highlighted with dashed ovals and Roman numerals, however, they do not form salt bridges.

Residues 637–639 of A and B subunits form the strand βU which is the central part of an antiparallel beta-sheet that also includes strands βJ (res 387–389) and βK (res 412–414) (Figs. 3b, c, 5a). However, strand βU is preceded by residues 621 to 632, which form different structures in the A and B subunits (Fig. 5a). The strand βU of the A1 subunit is inserted between the strands βJ and βK of the A2 subunit related by 72° counterclockwise rotation when looking from the inside of the particle (Fig. 5a). These strand insertions between adjacent A subunits propagate around the fivefold symmetry axis and may stabilize the decamer structure (Fig. 5a). Salt bridges at two positions stabilize the domain swap between A subunits. The salt bridges between Arg616 of the A1 subunit and Asp84 and Asp90 of the B1 subunit (Fig. 5b and Fig. 4j) stabilize the point where the C-terminal arm leaves the A1 subunit. The next set of salt bridges between Glu635 of the A1 subunit and Arg390 and Arg332 of the A2 subunit (Fig. 5b) stabilizes the point where the C-terminal arm of A1 inserts into the A2 subunit. In the B1 subunit, the C-terminus of which does not participate in domain swapping, no such interactions are present (Fig. 5).

The domain swaps between A subunits are not formed in L-A and *Trichomonas vaginalis virus 2*^16,22^. However, it is possible that decamers of capsid proteins of these viruses are stabilized by other structural features such as the “thumb” protrusions of *Trichomonas vaginalis virus 2*^22^. Since L-BC virus has smaller interfaces between capsid proteins than L-A and *Trichomonas vaginalis virus 2* (Supplementary Table 1), the domain swaps between A subunits of L-BC may be required to achieve the same stability of capsid protein decamers as those of the other viruses. A domain swap is also formed by the capsid proteins of the *Omono River Virus* (unclassified *Totivirus*-like virus). Unlike in L-BC, the domain swap involves the C-terminus of the B subunit, which interacts with two neighboring A subunits^23^.

### Putative cap-cleavage or cap-snatching active site

It has been shown that the homologous residues His154 of L-A and His156 of L-BC are involved in the binding of 7-methylguanosine (m7G) caps of the host mRNAs^47,49,50^. His156 of L-BC is located in a pocket on the outer surface of the L-BC capsid (Supplementary Fig. 5c, d). The putative decapping pockets of both L-A and L-BC have predominantly negative charge (Supplementary Fig. 5a, b). The covalent attachment of the eukaryotic m7G cap to His156 of L-BC virus was reported to depend on Mg^2+^ ions. We speculate that the ions may mediate the interaction of the negatively charged cleft with the negatively charged phosphate groups of mRNAs^50^.

### Structure of open particle

**Fig. 6:**
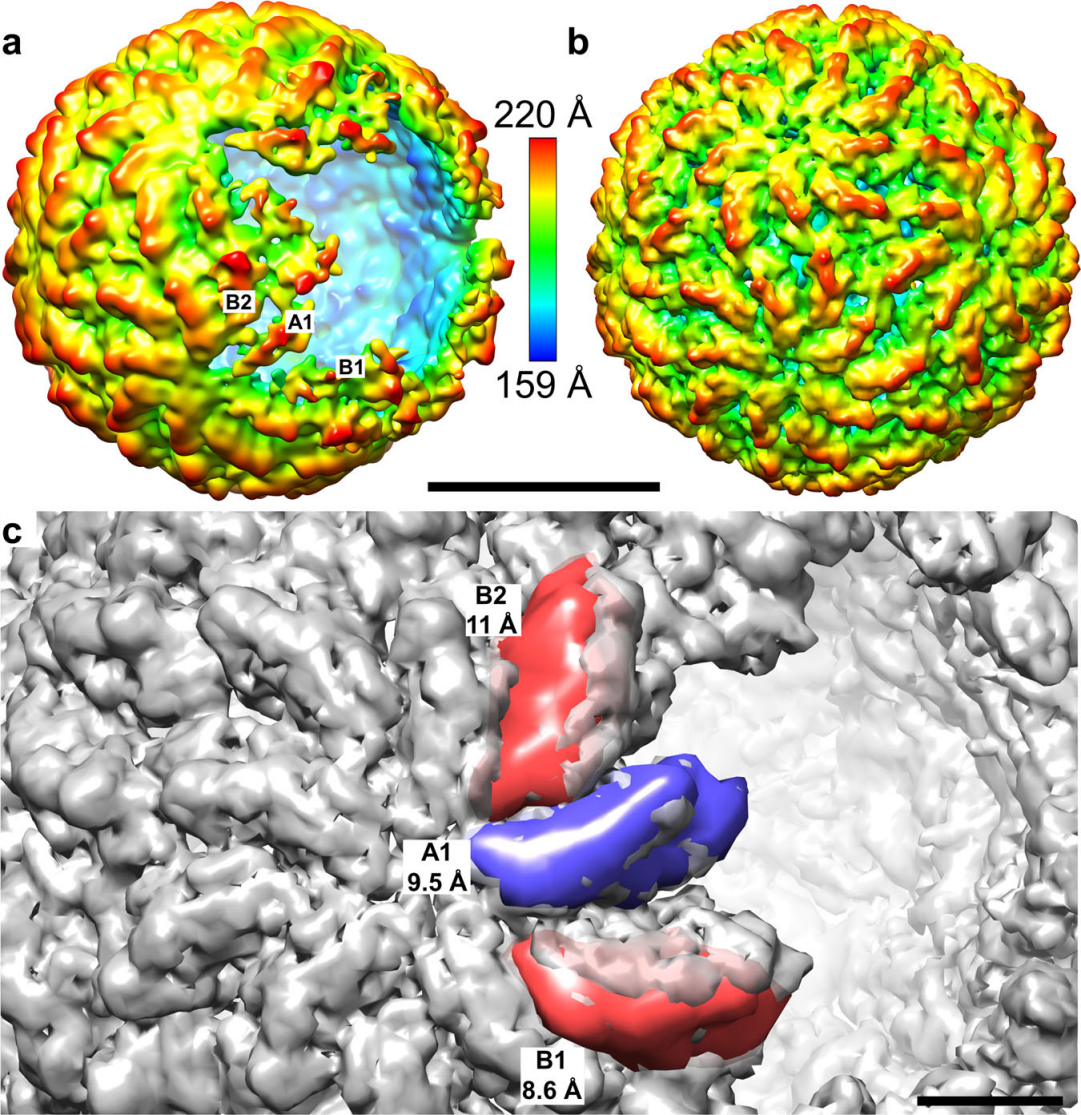
The structure of L-BC open particle. **a**, **b** Cryo-EM densities of open (**a**) and complete (**b**) L-BC particles rainbow-colored according to the distance of the surface from the particle center. Selected capsid proteins bordering the opening are labeled. Scale bar 200 Å. **c** Displacement of capsid proteins forming the edge of the opening. The cryo-EM reconstruction of the complete particle is shown as a gray semi-transparent surface. The density of a decamer of capsid proteins was removed from the complete particle to indicate the position of the opening. The colored surfaces show positions of the three subunits that form the edge of the hole in the open particle (the A subunit is in blue, B subunits in red). The indicated distances show the relative displacements of the corresponding subunits in the complete and open particles. The displayed densities were calculated from PDB structures fitted into the cryo-EM reconstruction of the open particle. Scale bar 50 Å. The map is contoured at 0.8 sigma for A and 2.0 sigma for B and C.

The two-dimensional classification of L-BC particle images identified a subpopulation of 9% of particles missing segments of their capsids (Fig. 1d). Asymmetric reconstruction of the open particles lacking a large segment of its capsid with the opening centered around a fivefold axis (Supplementary Fig. 6). The borders of the capsid opening are formed by poorly resolved density which may be caused by the limited number of images (1120) available for the reconstruction or by variations in the structure of individual open particles. Three-dimensional reconstruction of the open particles, with imposed fivefold symmetry, produced a 10 Å resolution capsid structure with one missing decamer of capsid proteins (Table 1 and Supplementary Fig. 1). The opening in the particle has a diameter of 16 nm (Fig. 6a). Overall, the structure of the open particle corresponds to that of virions and empty capsids (Fig. 6b); however, fifteen capsid proteins, two B and one A subunit from each of the five decamers that border the opening, have smeared densities indicating their high mobility. The three subunits are located further away from the particle center, and the B subunits are also shifted away from the hole. The total displacement of the border subunits is between 9 and 11 Å relative to their positions in the complete capsid (Fig. 6c). The changes in positions of the subunits forming the edge of the opening are enabled by their movement into the crevices and openings within the capsid.

### Incorporation of RdRp and genomes into *Totivirus* virions

Viruses L-A and L-BC have the same arrangement of open reading frames in their genomes^36,53^. Thus RdRp of L-BC is probably expressed as a C-terminal extension of the capsid by means of -1 ribosomal frameshifting facilitated by a putative slippery sequence GGATTTT (positions 1967–1973, NC_001641.1), which is found in the overlap between the two open reading frames (Supplementary Fig. 7). The probability of incorporating a certain number of capsid-RdRp proteins into a virion during assembly is described by the binomial distribution (1).

1 $$P=\frac{{(1-E)}^{D-E}{E}^{N}D!}{N!\left(D-N\right)!}$$

In the equation, *E* indicates the efficiency of ribosomal frameshifting, D is the number of protomers in the assembly (120 for a *Totivirus* virion, 10 for a decamer), and N is the number of capsid-RdRps per particle. The Eq. (1) assumes that the probabilities of incorporating a capsid protein and capsid-RdRp into a capsid are the same. However, the efficiency of the frameshifting is 1.9% in the L-A virus^40^. Therefore, particles with 1, 2, or 3 capsid-RdRp fusion proteins occur with 23, 27, and 20% probability, respectively. Moreover, using Eq. (1), it is possible to calculate the number of capsid-RdRp proteins in a decamer. In L-A virus, 82.5% of decamers are composed of only capsid proteins, 16% contain one RdRp, and the remaining 1.5% contain 2 or more RdRps. Increasing the probability of the frameshift producing capsid-RdRp to 4% or higher renders the L-A virus non-viable^40^. In such a case, 7.7% of decamers contained two or more capsid-RdRp proteins. When incorporated into a particle, decamers containing more than one capsid-RdRp protein may hamper transcription or destabilize the virion.

Assuming that the efficiency of ribosomal frameshifting is the same for L-A and L-BC viruses, 10% of particles should have no polymerase or genome. However, the purified L-BC sample contained 87% empty particles and only 4% virions (Fig. 1). This indicates that during the sample preparation, the fraction of empty particles was enriched or the genome release was induced. The observed particle damage could have been induced by the freeze-thaw cycle required for sample shipping or the low ionic strength of the buffer (20 mM Tris-HCl pH 7.5, 50 mM KCl, 10 mM MgCl_2_) used during particle vitrification. It was shown that exposing the L-A virus to 50 mM NaCl results in a 25% decrease in transcription activity^54^. In addition, this tendency of fungal dsRNA viruses to lose the genome has been described previously^34^. The low stability of dsRNA viruses that reside in the host cytoplasm may be due to their lack of need to protect their genomes in the extracellular environment, as is the case for viruses whose virions regularly infect cells.

So far, cryo-EM studies of totiviruses failed to resolve the polymerase inside the virus particle. The location of polymerase inside the particle is unlikely to be random, since its proper positioning and interactions with the inner capsid wall are essential for the regulation of transcription and mRNA translocation into cytoplasm^28–31^. However, localized refinements of the L-BC decamer followed by symmetry expansion and various classification approaches on both full and empty particles did not yield any inner density corresponding to the polymerase. This may be caused by variable positions of the RdRp domains among the RNA genome inside virions.

### L-BC capsid assembly

The domain swaps of βU strands between A subunits related by a fivefold axis and the existence of open particles indicate that capsids of L-BC virus are assembled from decamers. We speculate that the capsid assembly proceeds in three steps: (1) the formation of asymmetric AB dimers corresponding to the icosahedral asymmetric unit, (2) the formation of decamers from five AB dimers, and (3) the assembly of a capsid from 12 decamers. The conformation of the B subunit, with a βU strand forming a βJ-βU-βK sheet within the same chain, is probably the default arrangement of the capsid protein. The release of βU from its intramolecular βJ-βU-βK sheet may be induced by the interaction of the helix(ɑ2)-turn-helix(ɑ3) motif of the future B subunit with the residues 611–618 preceding the βU strand from the future A subunit upon the formation of AB dimers. The salt bridges between Asp90, Asp84 of B, and Arg616 of the A subunit (Fig. 5b) and a hydrogen bond between Gly92 of B subunit and Tyr623 of the A subunit may stabilize the C-terminus of the A subunit out of the βJ-βU-βK sheet. The free C-termini of A subunits may prime AB dimers for the decamer assembly.

The proposed three-step assembly pathway is similar to that of the *Bluetongue virus* (*Reoviridae*) subcore, which has the same type of symmetry as *Totivirus* capsids^29,55,56^. The formation of dimers and decamers of capsid proteins of *Bluetongue virus* was demonstrated experimentally by deleting the “dimerization domain” from the core protein, which prevented the association of decamers into subcore particles and promoted their accumulation in the cell^57^. Moreover, the subcore of *Bluetongue virus*, devoid of middle and outer shell layers, is transcriptionally active and infectious when introduced into the cell cytoplasm^58^, invoking parallels with totiviruses. Owing to their much more complicated virion structure, reoviruses require viroplasm, composed of non-structural proteins, to guide their particle assembly^59,60^. On the other hand, totiviruses are not known to code for any non-structural proteins, hence, their capsid assembly is likely to be stochastic.

The capsid shell immediately surrounding the genome has a unique but universally conserved arrangement among all dsRNA viruses (to the exclusion of *Birnaviridae* with an asymmetric inner core^61^), pointing to their common evolutionary origin^24,51^. Therefore, the assembly pathway of the shell may also be conserved. So far, the existence of decamer intermediates has only been experimentally verified for the *Bluetongue virus*^57^. In addition, it was also predicted for *Cytoplasmic polyhedrosis virus* (*Reoviridae*) and *Penicillium chrysogenum virus* (*Chrysoviridae*) based on capsid stabilization by protrusion proteins^62^ and contacts between asymmetric units^63^. We present the evidence for the decamer as an assembly intermediate of L-BC virus (*Totiviridae*). The only possible exception for the “assembly-by-decamer” rule is *Penicillium stoloniferum virus F* from the family *Partitiviridae*, which was predicted to assemble from 30 diamond-shaped tetramers^52^.

## Conclusions

Here we show that decamers of capsid proteins of L-BC virus are stabilized by domain swapping of the C-termini of A subunits located around icosahedral fivefold axes, and that capsids of 9% of particles in a purified L-BC sample were open and lacked one decamer of capsid proteins. Furthermore, we identified the asymmetric interactions necessary for the initiation of the domain swap and decamer formation. Based on these observations, we propose that the assembly of *Totiviridae* capsids is initiated by the formation of asymmetric capsid protein dimers that assemble into decamers, which are the building blocks of capsids. Low-abundance capsid-RdRp fusion proteins are incorporated into the capsid stochastically and enable the encapsidation of *Totivirus* genomes.

## Methods

### Yeast cell culture, particle purification, and vitrification

The yeast strain BY4741 ski3∆ (MATa *his3Δ1 leu2Δ0 met15Δ0 ura3Δ0* ski3∆ *ScV-LA-1* and *ScV-L-BC*), received from prof. Elena Servienė (Nature Research Center, Vilnius, Lithuania), was grown at 30 °C in 1% yeast extract, 2% peptone, and 2% glucose medium for 48 h. Cells were harvested by centrifugation at 5000 × *g* for 5 min at 4 °C, resuspended in lysis buffer (50 mM Tris-HCl pH 8.0, 200 mM NaCl, 2.5 mM EDTA, and 1 mM PMSF), and lysed by vigorous shaking with glass beads. Cell debris was removed by centrifugation at 10,000 × *g* at 4 °C for 30 min. L-BC particles were precipitated from the resulting supernatant by the addition of PEG-4000 to a final concentration of 5% (v/w), 500 mM NaCl, and incubation for 24 h at 4 °C. The precipitated L-BC particles were pelleted by centrifugation at 15,000 × *g* at 4 °C for 10 min. The pellet was re-suspended in the lysis buffer, dialyzed against the lysis buffer containing 50% glycerol, and stored at −20 °C. A centrifugal concentrator with a 100 kDa cut-off (Corning Spin-X UF) was used to remove glycerol, change the buffer to 20 mM Tris-HCl pH 7.5, 50 mM KCl, 10 mM MgCl_2_, and concentrate virus particles to 1.5 mg/ml. The L-BC virus sample (3.5 µl) was pipetted on a Quantifoil R 2/2 mesh holey carbon grid, blotted, and vitrified using a Vitrobot Mark IV (blot force 0, blot time 3 s, 4 °C, 100% humidity). Double sample application was performed to increase the amount of virus particles in holes.

### Data acquisition and processing

The dataset was acquired on a TFS Titan Krios operated at 300 kV, equipped with a Bioquantum 967 energy filter and Gatan K2 camera operating in counting mode. Each image was recorded as a movie over 6 s of exposure, resulting in a total dose of 36 e^−^/Å^2^. The nominal magnification was 130,000× resulting in a pixel size of 1.079 Å. The defocus range for the dataset was from −1.7 to −0.7 µm. SerialEM 3.7.14 software was used for the automated collection of 11,977 images. Movie frames were dose-weighted and aligned using MotioCorr2 1.4.0 to compensate for the beam-induced motion of the sample^64^ and the contrast transfer function (CTF) parameters were estimated using Gctf 1.06^65^. The dose-weighted sum of aligned frames was used for automated virus particle picking by crYOLO 1.7.5^66^ with a custom trained model and a detection threshold of 0.5, resulting in a total of 72,705 particles. Particle coordinates were passed to RELION 3.1 for image processing^67,68^. The particles were extracted with a box size of 560 pixels and subjected to reference-free two-dimensional classification. The resulting best classes corresponding to virions (1748 images), empty (38,763 images), and open (3812 images) particles were processed in parallel. The initial model of the L-BC virus capsid was generated *de novo* by stochastic gradient descent using the dataset of empty particles. Empty particle images were 3D-classified and filtered by the maximum value of probability distribution (50th percentile by rlnMaxValueProbDistribution) to obtain a homogeneous set of best-aligned particles (17,702 images). The RELION 3dautorefine procedure was used to reconstruct the full and empty particles with imposed icosahedral symmetry. To account for varying beam tilt during data acquisition, the particle images were split into nine optic groups according to the group of the beam-shifted acquisition area of the micrograph they originated from. Estimates of magnification anisotropy, beam tilt, and optical aberrations (up to 4th order) were done for each of the nine optic groups separately. Estimations of defocus parameters and b-factors were performed for individual particle images. The refinements were done iteratively (three rounds) following the scheme: 3dautorefine -> Ewald sphere correction -> masking -> CTF/aberration estimations. The resulting icosahedral reconstruction was postprocessed using the XMIPP local deblur tool (part of Scipion 2.0 package) which performs adaptive b-factor sharpening based on the local resolution of the map^69^. The final map resolution was determined using the FSC_0.143_ threshold criterion^70^.

### Open particle reconstruction

The 3D reconstruction of the open particle dataset (using 4× binned images, initiated with the icosahedral L-BC capsid filtered to a 30 Å resolution) with fivefold symmetry (C5) yielded a map with weak density on one of the capsid protein decamers positioned on the fivefold symmetry axis (oriented along the Z-axis), suggesting that the alignment of the missing decamers was imperfect. Subsequent masked 3D classification without alignment (--skip_align option in RELION) focused on the region of the front decamer resulted in two distinct classes: 2692 particles with a front decamer clearly present and 1120 particles without a front decamer. Further local C5 refinement of the minor class produced an open particle with no density of the front decamer. Attempts to enrich the open particle class by running classifications focused on 11 other decamers or applying an iterative refinement-classification approach focused on the missing decamer were unsuccessful. The average of two half-maps from the final reconstruction was low-pass filtered to 16 Å and multiplied by the mask representing an icosahedral particle without a front decamer. The processed map was subsequently used to rigid-body fit previously refined A and B capsid proteins. The subunits around the hole were fitted using the program Chimera 1.13^71^.

The particle orientations from the final C5 reconstruction were symmetry-expanded to C1 and subjected to C1 refinement with restricted searches for tilt- and psi-angles and full search for rot-angle using RELION 3.1. The symmetry-related variants of the particle orientation were then scored based on the goodness of fit to the resulting cryo-EM density map (rlnMaxValueProbDistribution value in RELION 3.1 data-star-file). For each particle image, the orientation with the highest score out of the five possible variants was retained. The restricted C1 refinement was repeated, starting from the best-selected particle orientations.

### Model building and refinement

The electron density map of an empty particle was cropped, normalized, rotated into 222 orientation, the map origin set to the center of the particle, and the crystallographic P23 space group applied. The initial model for the L-BC capsid protein was obtained from RaptorX Server^72^. The fit of the refined coordinates to the experimental density map was assessed and corrected manually using Coot 0.9^73^. Several rounds of real-space refinements in Phenix 1.19^74^ and reciprocal-space refinements in Refmac5^75^ were performed. The quality of the model was validated using MolProbity server^76^. Finally, to avoid clashes of residues between icosahedral asymmetric units, reciprocal-space refinement with 60 asymmetric units in the P23 map was performed using Refmac5 with the NCS constraints option. Structure visualization was performed in UCSF Chimera (1.15)^71^ and UCSF ChimeraX (1.2.5)^77^. The interfaces between capsid proteins were analyzed using the PISA EMBL-EBI web service^78^.

### Statistics and reproducibility

Cryo-EM reconstructions were performed according to the “gold-standard” approach used in structure determination^79,80^. Particles were randomly assigned the two subsets that were reconstructed independently.

### Reporting summary

Further information on research design is available in the Nature Research Reporting Summary linked to this article.

## Supplementary information

Supplementary Information

Reporting Summary

## Data Availability

The refined models and their corresponding electron density maps were deposited under the following PDB and EMD codes: empty particle 7QWX and EMD-14194, genome-containing virions 7QWZ and EMD-14195, open particle C5 reconstruction 7ZUF and EMD-14975, open particle C1 reconstruction 7ZTS and EMD-14963. The source cryo-EM movies were deposited to Electron Microscopy Public Image Archive (EMPIAR) under the following accession code: EMPIAR-11112.
