# Supplementary material for: Structures of L-BC virus and its open particle provide insight into Totivirus capsid assembly
Source: Commun Biol. 2022 Aug 20;5:847. doi: 10.1038/s42003-022-03793-z (PMC9391438; doi:10.1038/s42003-022-03793-z)
Supplement: Supplementary file 1 — Supplementary Information [file 42003_2022_3793_MOESM1_ESM.pdf]

Supplementary Information to

Structures of L-BC virus and its open particle provide insight into *Totivirus* capsid assembly

Short title: Virion structure of L-BC virus

Danyil Grybchuk[1], Michaela Procházková[1], Tibor Füzik [1], Aleksandras Konovalovas[2], Saulius Serva[2], Vyacheslav Yurchenko[3], Pavel Plevka[1]

1 - Central European Institute of Technology, Masaryk University, 62500 Brno, Czech Republic
2 - Department of Biochemistry and Molecular Biology, Vilnius University, 10257 Vilnius Lithuania
3 - Life Science Research Centre, Faculty of Science, University of Ostrava, 71000 Ostrava, Czech Republic

Corresponding author: pavel.plevka@ceitec.muni.cz

Supplementary figures

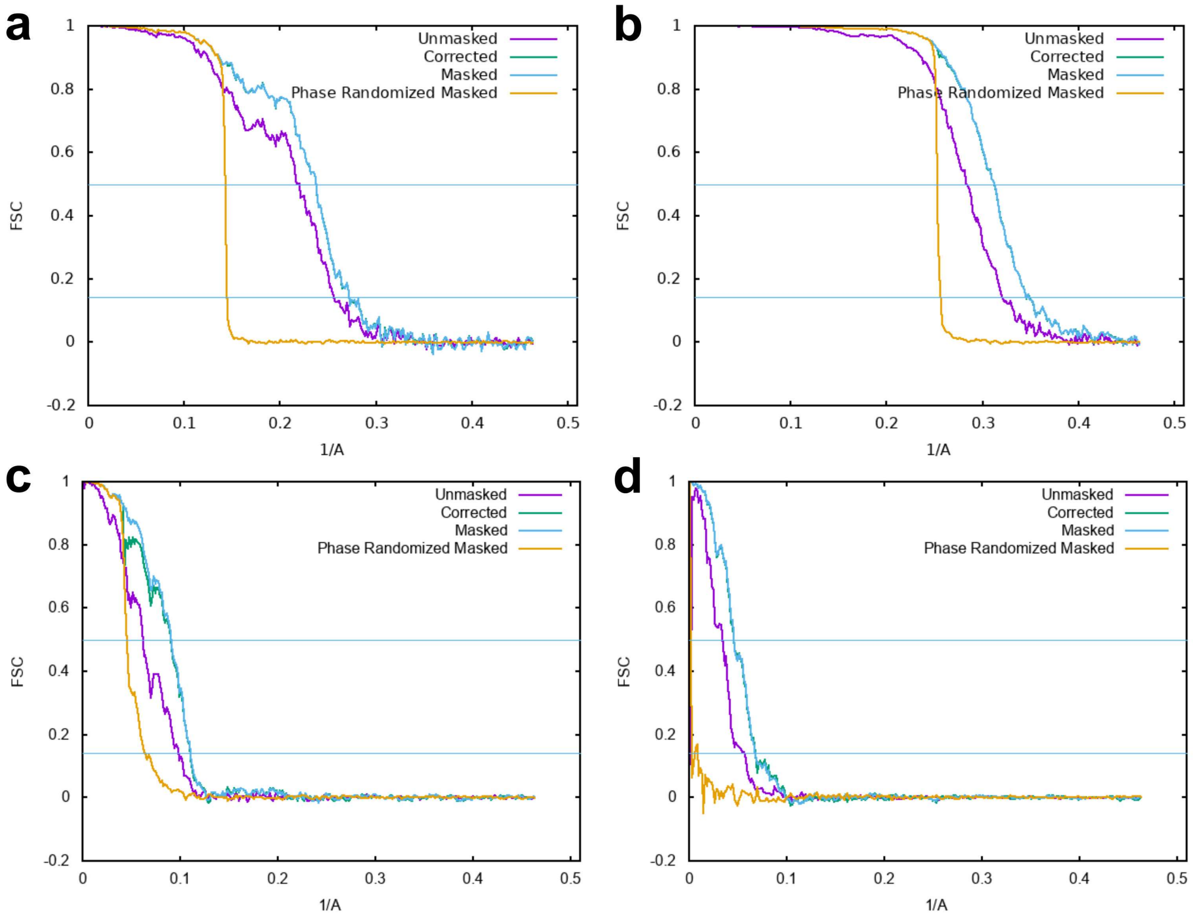

Supplementary Figure 1. FSC curves of cryo-EM reconstructions. (a) Full particle. (b) Empty particle. (c) Open particle C5 reconstruction. (d) Open particle C1 reconstruction.

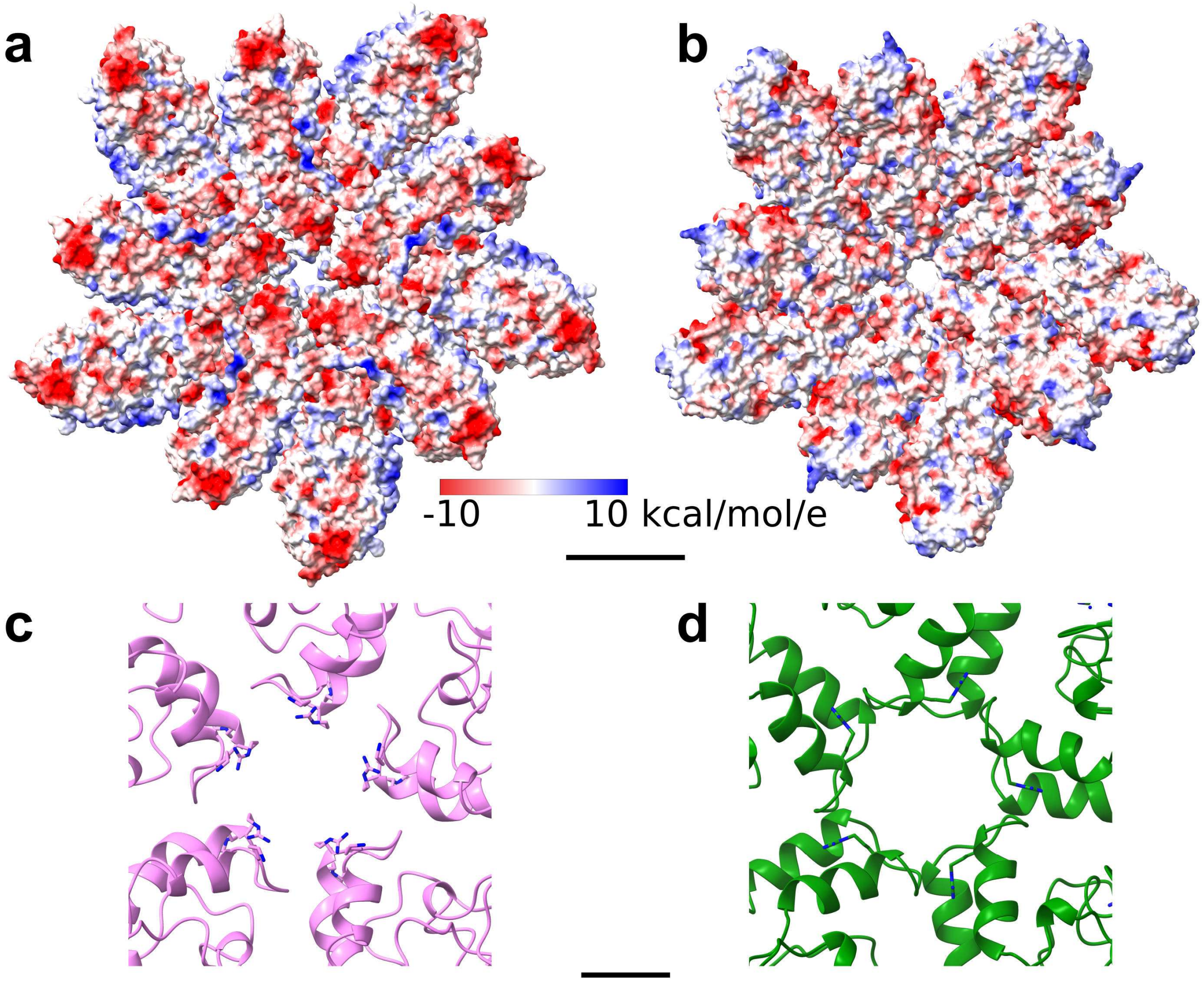

Supplementary Figure 2. Charge distribution at inner capsid surface and structure of pore along fivefold axis of L-BC and L-A viruses. (a-b) Decamers of capsid proteins of L-BC (a) and L-A (b) viewed from inside a capsid. The structures are shown as molecular surfaces colored according to the electrostatic surface potential. (c-d) Details of pores formed around fivefold axis of L-BC (c) and L-A (d) capsid. The structures of capsid proteins are shown in cartoon representation. Residues with positively charged side chains lining the pore are shown in stick representation. Scale bar represents 50 Å in (a) and (b) and 10 Å in (c) and (d).

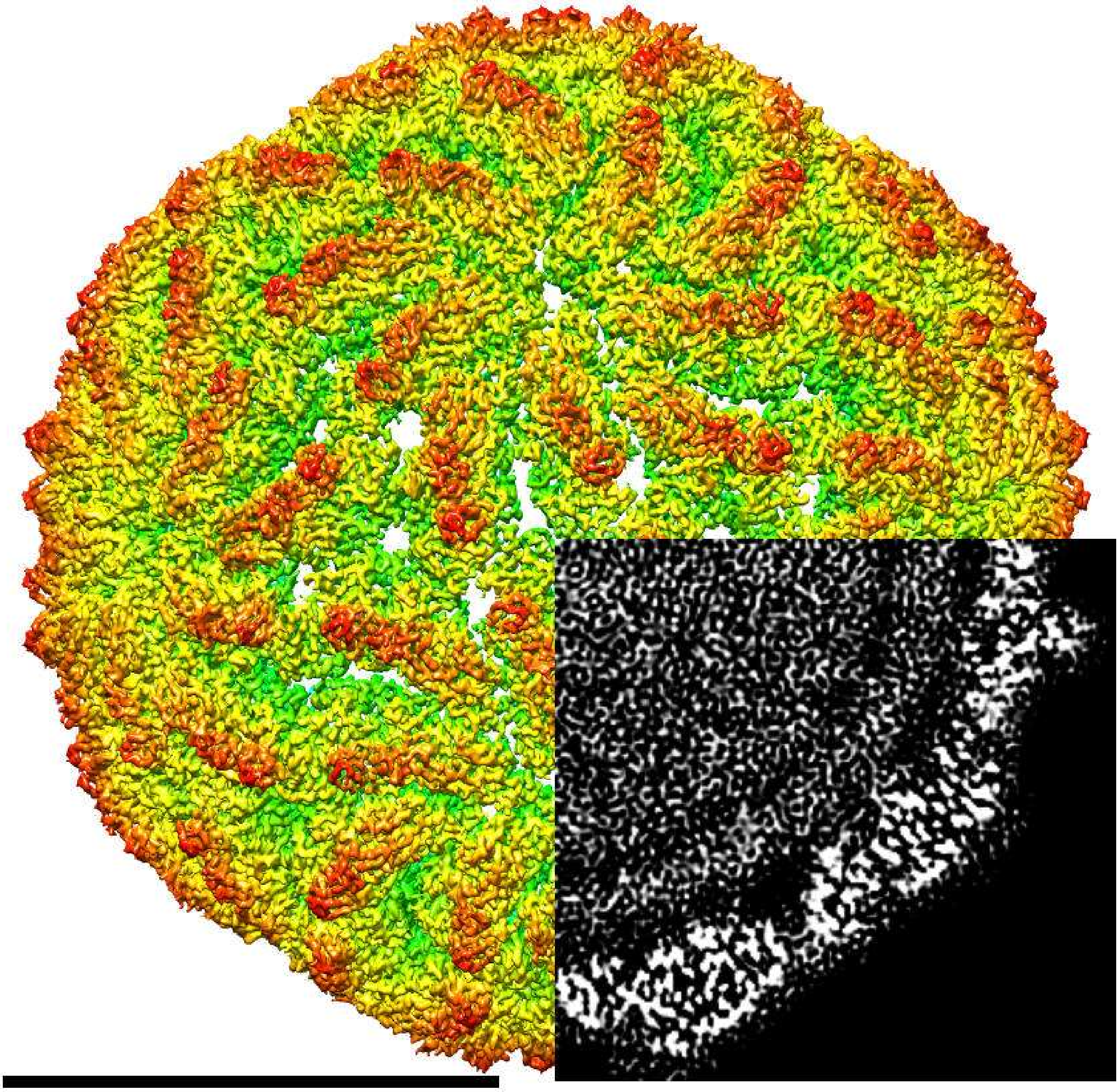

Supplementary Figure 3. Cryo-EM reconstruction of L-BC virion. Map of L-BC virion rainbow-colored based on the distance from the particle center. The inset is displaying a central slice of the cryo-EM density to show the distribution of the genome density inside the particle. Scale bar 200 Å. The map is contoured at 2.6 sigma.

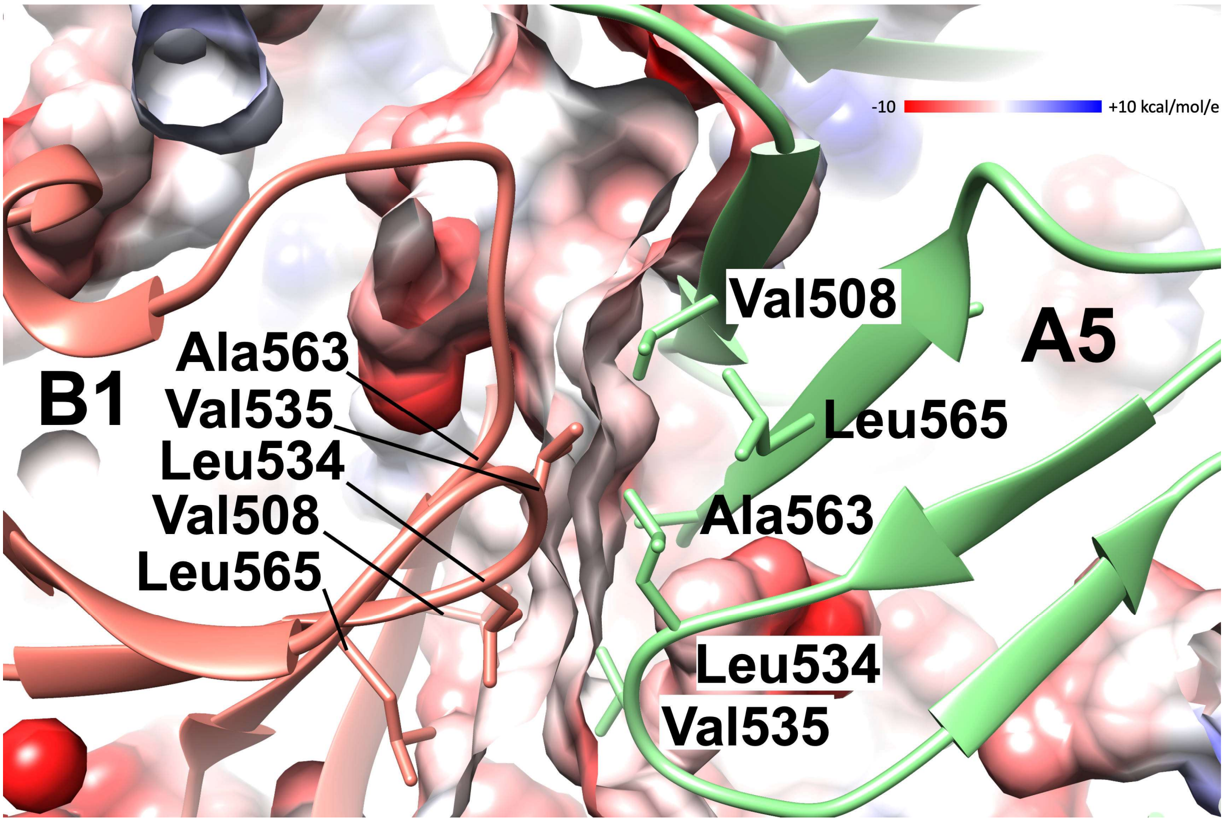

Supplementary Figure 4. Interactions between A and B subunits related by quasi-twofold axis are mediated by residues Val508, Leu534, Val535, Ala563, and Leu565. The structures of the interacting A and B subunits are shown in cartoon representation with selected residues shown in stick representation. Molecular surfaces are colored according to their electrostatic potential to show their predominantly neutral charge.

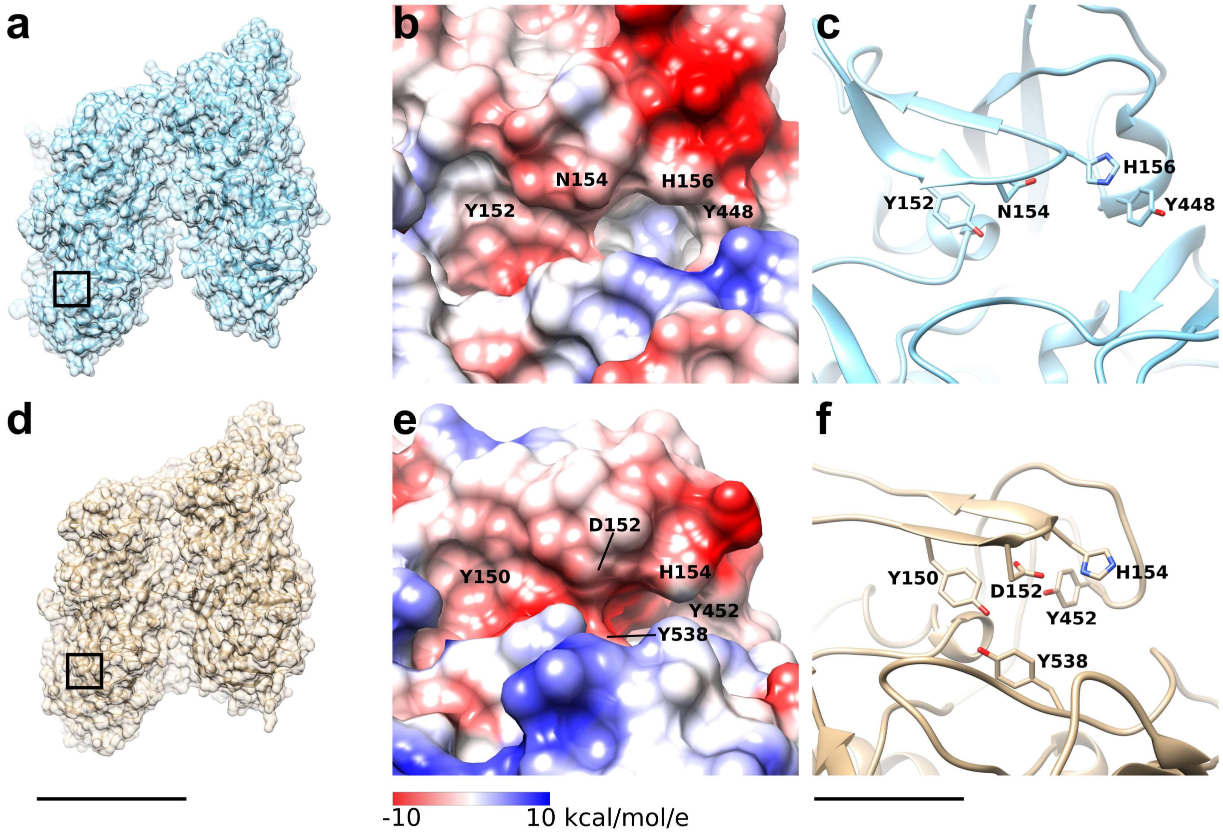

Supplementary Figure 5. Comparison of putative decapping pocket in B subunit of L-BC (a-c) and L-A (d-f) viruses. (a, d) Molecular surface representation of icosahedral asymmetric units of L-BC (a) and L-A (d). The position of the decapping pocket in the B subunit is marked with a rectangle. (b, e) Molecular surface representation of decapping pocket of L-BC (b) and L-A (e). The surfaces are colored according to their electrostatic surface potential. (c, f) Cartoon representation of decapping pocket regions of L-BC (c) and L-A (f). Side chains of conserved residues essential for cap-snatching are shown in stick representation. Scale bars represent 50 Å in (a) and (d), 10 Å in (b, c, e, and f).

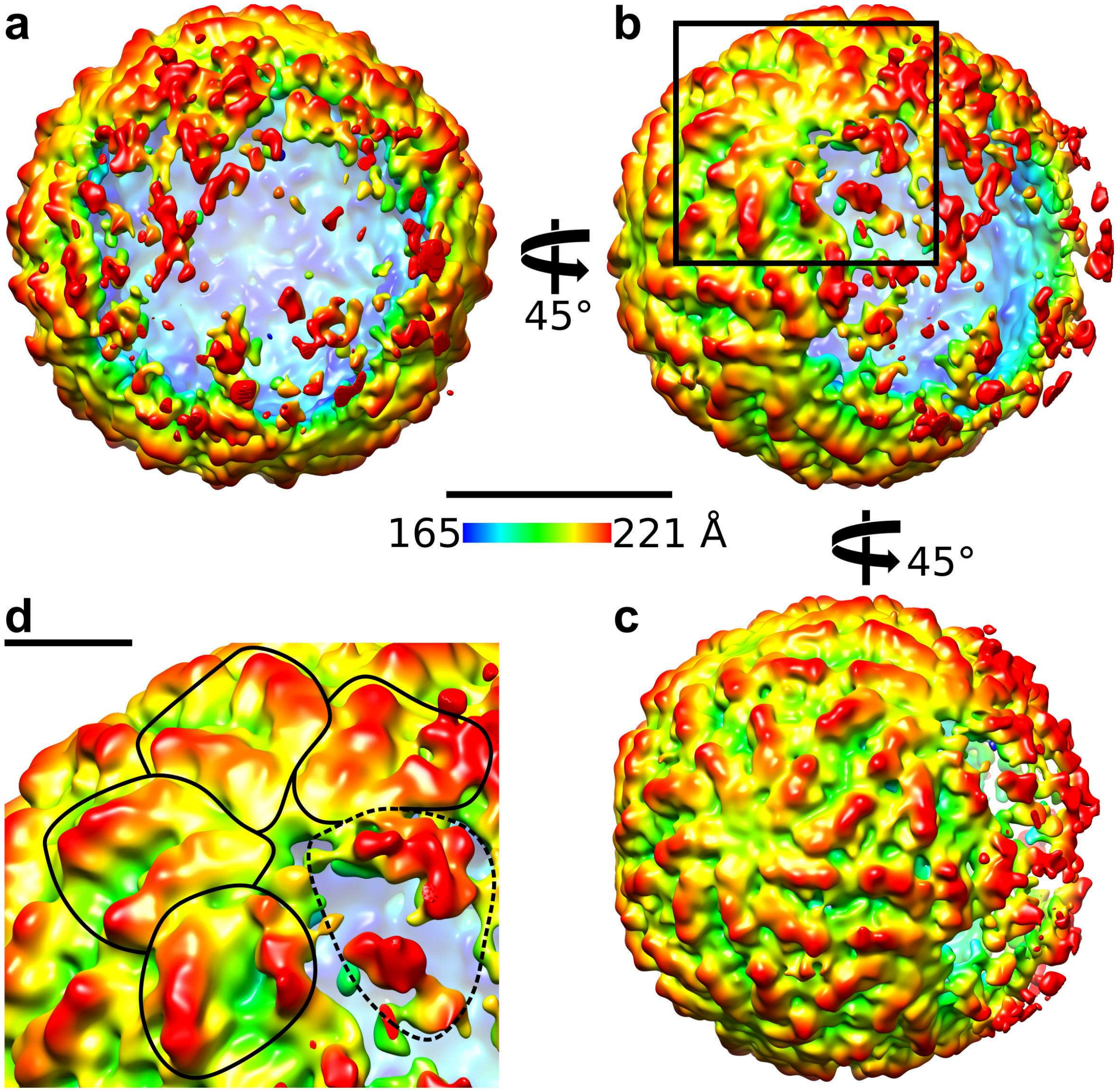

Supplementary Figure 6. Asymmetric reconstruction of an open particle of L-BC. (a-b) Surface representation of asymmetric cryo-EM reconstruction of the open particle. The surface is rainbow colored based on the distance from the particle center. The map is displayed at the 1 sigma contour level. The rectangle in (b) indicates the detail shown at higher magnification in panel (d). (d) Four asymmetric units at the border of the capsid opening are clearly resolved (indicated by black outlines), whereas the density of the fifth asymmetric unit is less well defined (indicated by black dashed outlines). Scale bars represent 50 Å in (a-c), and 10 Å in (d).

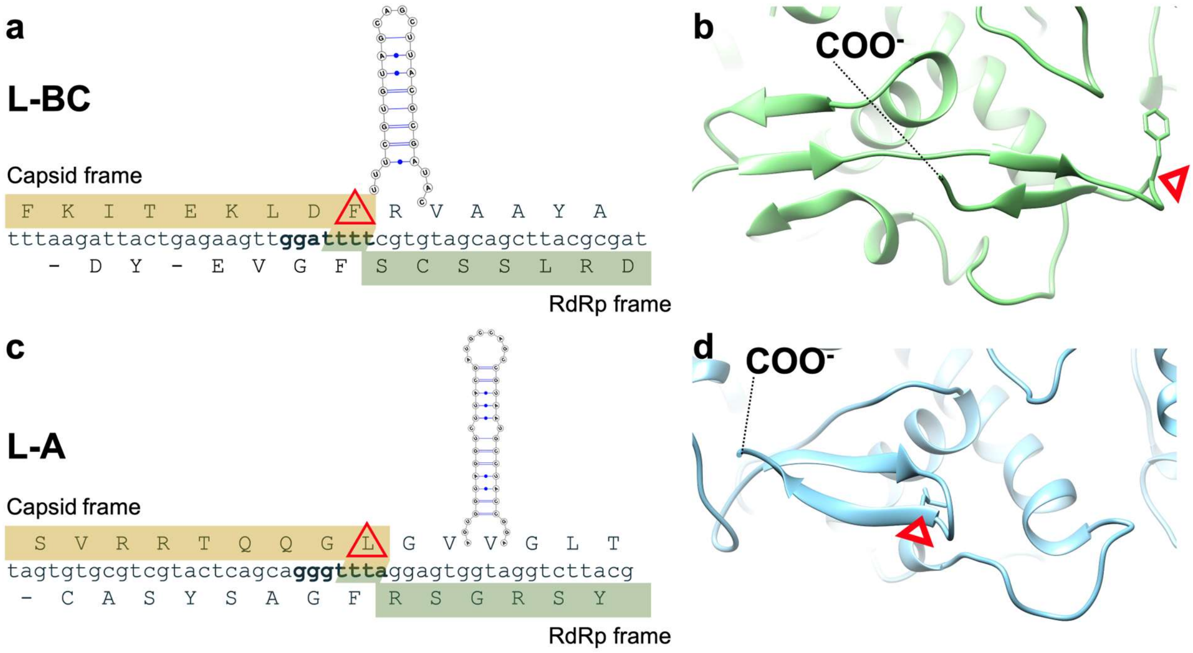

Supplementary Figure 7. Comparison of putative -1 ribosomal frameshifting RNA sequences and positions of capsid-RdRp linkers in structures of L-BC and L-A subunits. (a, c) RNA sequences that enable -1 ribosomal frameshifting in L-BC (a) and L-A (c) viruses[1]. The frameshifting sequence is indicated in bold, the last common residue of the capsid protein and capsid-RdRp fusion is indicated with a red triangle. The RNA hairpin structures immediately downstream of the slippery site were predicted with the software IPknot[2]. (b, d) Positions of last common residue of capsid protein and capsid-RdRp fusion within capsid protein structure are indicated with red triangles in L-BC (b) and L-A (d). Side chains of these residues are shown in stick representation.

Supplementary tables

Supplementary table 1. Largest buried surface areas between A and B subunits of structurally characterized totiviruses

	Buried surface area (Å^2)	
Virus	A1/B1	A2/B1
L-BC	1350	1350
L-A	1550	1400
LRV1	2050	1150
TVV2	1500	1250

Supplemental references

1. Dinman, J. D., Icho, T. & Wickner, R. B. A -1 ribosomal frameshift in a double-stranded RNA virus of yeast forms a gag-pol fusion protein. *Proc Natl Acad Sci U S A* **88**, 174–178 (1991).

2. Sato, K., Kato, Y., Hamada, M., Akutsu, T. & Asai, K. IPknot: fast and accurate prediction of RNA secondary structures with pseudoknots using integer programming. *Bioinformatics* **27**, i85-93 (2011).
